# Role of Actionable Genes in Pursuing a True Approach of Precision Medicine in Monogenic Diabetes

**DOI:** 10.3390/genes13010117

**Published:** 2022-01-09

**Authors:** Antonella Marucci, Irene Rutigliano, Grazia Fini, Serena Pezzilli, Claudia Menzaghi, Rosa Di Paola, Vincenzo Trischitta

**Affiliations:** 1 Research Unit of Diabetes and Endocrine Diseases, Fondazione IRCCS Casa Sollievo della Sofferenza, San Giovanni Rotondo, 71013 Foggia, Italy; g.fini@operapadrepio.it (G.F.); s.pezzilli@css-mendel.it (S.P.); c.menzaghi@operapadrepio.it (C.M.); r.dipaola@operapadrepio.it (R.D.P.); 2 Pediatric Unit, Fondazione IRCCS Casa Sollievo della Sofferenza, San Giovanni Rotondo, 71013 Foggia, Italy; i.rutigliano@operapadrepio.it; 3 Department of Experimental Medicine, Sapienza University, 00161 Rome, Italy

**Keywords:** actionable genes, monogenic diabetes, MODY, syndromic diabetes, precision medicine, individual intervention

## Abstract

Monogenic diabetes is a genetic disorder caused by one or more variations in a single gene. It encompasses a broad spectrum of heterogeneous conditions, including neonatal diabetes, maturity onset diabetes of the young (MODY) and syndromic diabetes, affecting 1–5% of patients with diabetes. Some of these variants are harbored by genes whose altered function can be tackled by specific actions (“actionable genes”). In suspected patients, molecular diagnosis allows the implementation of effective approaches of precision medicine so as to allow individual interventions aimed to prevent, mitigate or delay clinical outcomes. This review will almost exclusively concentrate on the clinical strategy that can be specifically pursued in carriers of mutations in “actionable genes”, including *ABCC8*, *KCNJ11*, *GCK*, *HNF1A*, *HNF4A*, *HNF1B*, *PPARG*, *GATA4* and *GATA6*. For each of them we will provide a short background on what is known about gene function and dysfunction. Then, we will discuss how the identification of their mutations in individuals with this form of diabetes, can be used in daily clinical practice to implement specific monitoring and treatments. We hope this article will help clinical diabetologists carefully consider who of their patients deserves timely genetic testing for monogenic diabetes.

## 1. Introduction

Precision medicine is meant as the attempt to provide the most specific management (i.e., prediction, prevention, diagnosis, follow-up and treatment) for subgroups of individuals who share similar features such as those of epidemiological, phenotypic, clinical and molecular origin. In the last few years, genome and exome sequencing have helped identify genetic variants which shape the risk of various diseases. Some of these variants are harbored by genes whose altered function can be tackled by specific approaches, so as to allow individual interventions aimed to prevent, mitigate or delay clinical outcomes (“actionable genes”) [1].

Diabetes mellitus has a current prevalence of 463 million (equivalent to 9.3% of the world population) that is expected to raise to 578 million by the year 2030 [2]. Diabetes imposes heavy burdens on health care systems, patients, and their families and is associated with serious economic implications, making any effort to tackle it of paramount importance [3]. As much as 95% of patients with diabetes are affected by either type 1 (approximately 10%) or type 2 diabetes, both typical examples of complex diseases based on the interaction between environmental factors and a predisposing genetic background [4]. Conversely, a small proportion of patients with diabetes (up to 5%), which, however, given the huge number of affected individuals is not trivial in absolute terms, is affected by monogenic forms of the disease [4,5]. Of interest several actionable genes that cause monogenic diabetes have been described and this allows the implementation of effective approaches of precision medicine in carrier patients [6,7].

This review will offer a brief overview of our present knowledge of the actionable genes that cause monogenic diabetes. For each of them we will provide a short background on what is known about gene function and dysfunction. Then we will discuss how the identification of their mutations in individuals with this form of diabetes can be used in daily clinical practice to implement specific monitoring and treatments.

## 2. Monogenic Diabetes

Monogenic diabetes is a genetic disorder caused by one or more variations in a single gene. It encompasses a broad spectrum of heterogeneous conditions, including neonatal diabetes, maturity onset diabetes of the young (MODY) and syndromic diabetes [8], affecting 1–5% of patients with diabetes [5,9]. These forms of diabetes have either dominant or recessive or non-Mendelian inheritance. To date, 40 different subtypes of monogenic diabetes have been identified [5]. By far, the most frequent form of monogenic diabetes is MODY, which according to its historical definition [10,11] is inherited in an autosomal dominant manner and occurs in lean individuals before the age of 25 years, with no need of insulin therapy. This strict definition is now outdated, since MODY also occurs in middle age and/or overweight/obese patients and may need insulin as the most appropriate treatment [12]. Mutations in fourteen different genes have been so far reported to cause several MODY subtypes (OMIM # 606391), the most common of which are due to mutations in *GCK*, *HNF1A*, *HNF4A* and *HNF1B* [7].

A well-known additional form of monogenic diabetes is neonatal diabetes, a rare disorder characterized by marked insulin-requiring hyperglycemia within the first 6 months of life [13]. Between 50 to 60% of cases resolve within 18 months and are, therefore, termed transient neonatal diabetes. The remaining patients require insulin treatment for life and are termed permanent neonatal diabetes [14]. Approximately 20–30% of patients with neonatal diabetes also present neuro-motor developmental delay [15,16,17]. Additional neurological implications including autism, attention deficit hyperactivity disorder, anxiety, sleep disorders and learning difficulties with impaired attention and memory [18,19,20,21,22,23] have been also described.

Finally, very rare forms of monogenic diabetes are those presenting in combination with several additional extra-pancreatic abnormalities (i.e., syndromic diabetes) which are autosomal, X-linked, recessively and dominantly inherited or due to mitochondrial mutations. Syndromic forms of diabetes can derive from severe defect of either insulin secretion (e.g., Wolfram syndrome, Wolcott–Rallison syndrome, thiamine-responsive megaloblastic anemia, mitochondrial mutations) or insulin action (i.e., insulin receptor mutations, Bardet–Biedl syndrome, Alstrom syndrome, Berardinelli–Seip congenital lipodystrophies) [5,24].

In general, a monogenic form of diabetes should be suspected either in children or in young individuals with diabetes when hyperglycemia ensues with no typical features of type 1 or type 2 diabetes [4,25]. These includes some degree of ketoacidosis and especially the ineludible need of insulin treatment for type 1 diabetes. At variance, the typical characteristics of type 2 diabetes are the presence of obesity and other comorbidities clustering in the metabolic syndrome, including which hypertension and atherogenic dyslipidemia are the most common. Overall, the great heterogeneity of all forms of hyperglycemia, makes sometimes difficult to differentiate monogenic diabetes from the type 1 and type 2 diabetes from a simple clinical point of view. It is therefore very useful that, in suspected patients, the simultaneous analysis of multiple genes by NGS, currently available in many contexts, gives the possibility of a rapid and precise molecular diagnosis. 

Since several excellent comprehensive reviews have been recently published on both molecular and clinical aspects of monogenic diabetes [5,8,9], this article will almost exclusively concentrate on the clinical strategy that can be specifically pursued in carriers of mutations in actionable genes, including *ABCC8*, *KCNJ11*, *GCK*, *HNF1A*, *HNF4A*, *HNF1B*, *PPARG*, *GATA4* and *GATA6* [6] see Table 1.

## 3. ABCC8 and KCNJ11

*ABCC8* and *KCNJ11* encode respectively four sulfonylurea receptor 1 (SUR1) and four inward rectifier potassium channel Kir6 (Kir6.2) subunits both belonging to the ATP-sensitive potassium (K_ATP_) channel in the pancreatic β-cells [26,27,28]. K_ATP_ channel links cellular glucose metabolism to electrical activity of the plasma membrane thereby regulating insulin secretion [27,29,30,31]. In details, at sub-stimulatory glucose concentration, the K_ATP_ channel is open, thus permitting the efflux of K^+^ from the β-cell that eventually causes membrane hyperpolarization. Channel opening is the result of a fine regulation and activation exerted by the binding of intracellular MgADP [27,32]. Conversely, when blood glucose level rises, β-cells metabolize glucose, converting ADP to ATP, which then binds to and closes the K_ATP_ channel causing membrane depolarization. This in turn activates voltage-dependent calcium channels, allows Ca^+^ influx into the cell and finally triggers insulin granule release [27].

More than 700 *ABCC8* and 200 *KCNJ11* pathogenic or likely pathogenic mutations have been reported [33]. As described below, mutations in these two genes result in a variety of phenotypes depending on the hyper- or hypo-activity of the K_ATP_ channel they induce. 

*ABCC8/KCNJ11* gain-of-function (GOF) mutations cause the permanent opening of the K_ATP_ channel [34], and thus reduce insulin secretion and eventually cause hyperglycemia with a wide spectrum of diabetes phenotypes, ranging from neonatal diabetes to MODY to adult-onset diabetes [35,36,37,38]. In *ABCC8* about 100 dominant and 100 recessive activating mutations have been described [39,40] to cause neonatal diabetes. Conversely, nearly 100 dominant and only one recessive activating *KCNJ11* mutations have been linked to neonatal diabetes [15,41]. About 60% of dominant mutations causing neonatal diabetes occur “de novo”; a germline mosaicism has been also observed in some families [42,43].

In contrast, far fewer MODY families with heterozygous *ABCC8* [44,45,46] and *KCNJ11* [47,48] mutations have been described worldwide.

*ABCC8/KCNJ11* loss-of-function (LOF) mutations affect K_ATP_ channel by both preventing its posttranscriptional moving to the plasma membrane or by reducing its responsiveness to MgADP activation. These mutations, mostly observed in *ABCC8* (nearly 600 vs. approximately 100 in *KCNJ11*) [49,50,51,52], are predominantly recessive inherited [53] and account for 36–70% cases of congenital hyperinsulinemic hypoglycemia (HH) [54,55], which is not the focus of this article and which has been discussed in details elsewhere [56,57]. 

### ABCC8/KCNJ11 Actionability

When a diagnosis of neonatal diabetes due to a K_ATP_ channel activating mutation is made, sulphonylureas is the treatment of choice, being effective as monotherapy in most of these patients. Sulphonylureas bind specifically to the SUR1 subunit and close the K_ATP_ channel via an ATP-independent mechanism, thus bypassing the genetic defect on *ABCC8*/*KCNJ11*h [58]. In fact, the great majority of mutation carrying individuals treated with insulin can be shifted to sulphonylureas, with a significant improvement of quality of life and disease management [59,60]. Though generally very good, the efficacy of this treatment seems to be partly determined by both the type of mutation and the duration of diabetes [61,62]. 

Despite the relatively high doses of sulfonylureas used in these patients, no episodes of hypoglycaemia have been reported during a long-term follow-up while persistent efficacy and safety of the treatment was described [63].

Moreover, in some patients with intermediated neuro-motor developmental delay, high dose of sulphonylureas initially improves the neurological impairment [18,21,63,64,65].

During pregnancy, the benefit of sulphonylureas treatment is dependent of the combination of *ABCC8* or *KCNJ11* genotype in the mother and the fetus:

(a) If the mother carries an *ABCC8* or *KCNJ11* activating mutation, and the fetus has a normal genotype, sulfonylureas therapy can result in excessive insulin secretion that induce macrosomia and neonatal HH in the baby [66,67,68]; when ultrasound monitoring results suggest this possibility, transfer from sulphonylureas to insulin is recommended [69].
(b) If both the mother and the fetus carry an *ABCC8* or *KCNJ11* activating mutation, babies benefit from early exposure to sulphonylureas treatment which prevents the low birth weight caused by reduced insulin secretion in the uterus [40,59]; sulphonylureas treatment should be, therefore, continued at the lowest dose required to obtain the optimal glycemic control [69].

Coincidentally, because of reduced insulin secretion in the uterus birth weight will be low also when only the fetus has an activating *ABCC8* or *KCNJ11* mutation (e.g., either inherited from the father or because of a “de novo” mutation) [70].

When one of the above conditions is known or suspected (e.g., father known to carry an activating *ABCC8* or *KCNJ11* mutation), ultrasound should be performed every two weeks from the 26th week of gestation to strictly follow fetal growth [71]. 

## 4. GCK

Glucokinase (GCK) is one of four members of hexokinase enzymes family [72,73]. GCK acts in the first reaction of glycolytic pathway converting glucose in glucose-6-phosphate. This will be completely metabolized through the next glycolytic steps and eventually increase intracellular ATP concentration, thus inducing pancreatic β-cells insulin secretion. In fact, GCK is considered the glucose sensor in β-cells, with the rate of glucose phosphorylation being directly related to plasma glucose concentration in the physiological range [73]. In addition to pancreatic islets, GCK is expressed in several tissues such as brain, liver and endocrine cells of the gut [74]. To date, over 900 *GCK* variants have been identified spanning all over the gene (e.g., missense/nonsense mutations, splicing mutations, small and gross deletions, insertions). *GCK* mutation should be suspected in the presence of blood glucose levels in the range of 5.5–8.0 mmol/l and a relative low increment at the oral glucose tolerance test (OGTT) [75]. According to the type of mutation (e.g., heterozygous or homozygous and LOF or GOF), different phenotypes can ensue.

*GCK* heterozygous LOF mutations have been associated with a reduced GCK activity that alters the glucose threshold for stimulating insulin secretion and eventually a mild form of fasting hyperglycemia present from birth and named as GCK-MODY (traditionally known as MODY2). Patients with GCK-MODY are also characterized by reduced hyperglycemia-induced inhibition of hepatic glucose output and decreased post-prandial liver glycogen synthesis. Despite lifelong hyperglycemia, GCK-MODY phenotype is characterized by a very low prevalence of microvascular and macrovascular complications [76].

Conversely as described below, pregnancy management is particularly important in patients with GCK-MODY [77,78]. 

*GCK* homozygous or compounds heterozygous LOF mutations usually cause neonatal diabetes, although few carriers of such mutations have been reported to be indistinguishable from those with typical GCK-MODY [79,80].

*GCK* heterozygous *GOF* mutations cause HH due to increased glucose-GCK affinity that causes excessive insulin secretion not commensurate with blood glucose levels [74,81]. Details on HH, which is not the focus of this review, have been already extensively discussed [56,57].

### GCK Actionability

With the exception of pregnancy, GCK-MODY patients do not need treatment because its mild hyperglycemia is not progressive. Only diet and lifestyle interventions should, therefore, be recommended [76,82].

In order to avoid abnormal fetal growth, hyperglycemia in women with GCK-MODY should be treated or not, according to the *GCK* genotype of both the mother and the fetus as it follows:

(a) If the mother carries a *GCK* mutation a non-mutated fetus senses maternal hyperglycemia and consequently increases insulin secretion that, in turn, causes macrosomia (suggested by accelerated fetal growth at ultrasound and resulting in a final birth weight increased by 550–700 g) [83,84]. In these cases, insulin treatment of the pregnant mother is necessary.
(b) If both the mother and the fetus carry a *GCK* mutation, insulin treatment is not recommended because fetal growth will be normal [72].
(c) If only the fetus has a LOF *GCK* mutation no treatment is needed, but usually the babies have reduced birth weight (by approximately 400 g) due to reduced insulin secretion in the uterus [83,84].

When one of the above conditions is known or suspected (e.g., father known to carry a LOF *GCK* mutation), ultrasound should be performed every two weeks from the 26th week of gestation to strictly follow fetal growth [71]. 

## 5. Hepatocyte Nuclear Factors (HNF) Family 

Hepatocyte nuclear factors (HNFs), which are classified into 4 families, were first identified as liver-enriched transcription factors and are known to be important regulator of pancreas, kidney and liver development and/or function [85,86,87]. LOF mutations in *HNF1A*, *HNF1B* and *HNF4A*, all leading to defective insulin secretion and reduced β-cells mass, cause some forms of MODY possibly associated with kidney and/or liver abnormalities [88].

For all these genes clinical phenotype of the mutation carriers is highly variable from one family to another and also within the same family [89].

### 5.1. HNF1A 

Until now more than 400 different variants [90] spanning from the *HNF1A* promoter to the 3′UTR region (e.g., missense mutations, frame shift, nonsense, splicing mutations, in-frame amino acid deletions, insertions, duplications or partial and whole-gene deletions) have been described, many of which occurring in exon 2 and 4 [90,91]. *HNF1A* heterozygous and homozygous [92] LOF mutations cause HNF1A-MODY (MODY3) [93,94,95], by impairing the transcriptional activity of the gene which, in turn, affects, several target proteins involved in glucose metabolism, insulin secretion [96,97,98] and cell proliferation [99,100,101]. Some of these genes are implicated in glycolysis and ATP production which is blunted by LOF *HNF1A* mutation [94,102,103,104].

HNF1A-MODY manifests, usually, in the first 2–3 decades of life with mild symptoms (polyuria, polydipsia) or as asymptomatic postprandial hyperglycemia, without ketosis or ketoacidosis. Fasting glucose may be normal at the disease’s onset and increases gradually with age, while 2-h glucose at the oral glucose tolerance test (OGTT) is frankly increased (4.4–5 mmol/l above the normal range) [105]. Insulin deficiency is progressive, C-peptide values are lower than in healthy individuals, but generally higher than in type 1 diabetes [105]. 

In kidneys, *HNF1A* LOF mutations reduce the threshold of glucose reuptake from the glomerular filtrate [106]. As a consequence, carriers of *HNF1A* mutations show glycosuria [105] that begins several years before hyperglycemia and is likely due to down regulation of *SLC5A2* [106] which encodes SGLT2, a sodium-dependent glucose transporter known to play a major role in renal glucose reabsorption. Liver adenomatosis may also be present [107,108,109,110]. Finally, increased fatty acid synthesis and altered transportation and eventually lipid accumulation into liver cells have been described [111].

In individuals with poor glycemic control, chronic microvascular complications (retinopathy, nephropathy and neuropathy) are common and ketoacidosis can develop [112,113]. The risk of hypertension and ischemic heart disease are similar to what observed in type 1 diabetes, much rarer than in type 2 diabetes, but certainly increased than in healthy controls [114]. Children exposed to high glucose levels in the uterus who inherit *HNF1A* mutation from their mother have an earlier onset of diabetes (5–10 years before) than those who inherit the mutation from their father [115].

#### HNF1A Actionability

Patients with HNF1A-MODY are sensitive to low-dose sulfonylureas [116,117]. In fact, SUR1 is downstream the HNF1A effects on glycolysis and mitochondrial ATP production. Sulphonylureas, therefore, bypass the β-cell defects induced by *HNF1A* mutations [107], so as to properly activate the downstream preserved machinery which is perfectly able to promote insulin secretion. Sulphonylureas are, therefore, the first-choice treatment in patients with HNF1A-MODY. Since in children and in adolescents sulfonylureas are not yet licensed, the use of meglitinides can be considered [118,119,120]. 

Sulfonylureas are usually effective for several decades, but in patients with severe insulin deficiency or after a long duration of diabetes (>11years), insulin treatment may eventually become necessary, either alone or on top of sulfonylureas [121]. 

GLP-1 Receptor Activation (GLP1-RA) stimulates insulin secretion bypassing the genetic HNF1A defect through the elevation of cyclic adenosine monophosphate (cAMP) and activation of protein kinase A [122,123,124]. GLP1-RA has been, therefore, recently suggested as an add-on treatment in patients who do not achieve optimal glycemic control with sulfonylurea or who experience frequent hypoglycemic events [125,126,127,128].

Although rare cases of macrosomic *HNF1A* mutation carriers presenting with neonatal transient HH that resolve with age have been described [129,130], *HNF1A* mutations do not generally influence birth weight because insulin secretion in the uterus remains normal [69,131]. Therefore, if only the fetus is suspected to carry an *HNF1A* mutation (e.g., father known to be mutated) no treatment is needed [69]. As far as the optimal treatment of gestational hyperglycemia is concerned, insulin is necessary if only the mother carries an *HNF1A* mutation [69,132]. 

### 5.2. HNF4A

*HNF4A* heterozygous LOF mutations are 10 times less frequent than those harbored by *HNF1A*. Until now more than 100 different variants [90] spanning the whole gene (e.g., missense mutations, frame shift, nonsense, splicing mutations, in-frame amino acid deletions, insertions, duplications or partial and whole-gene deletions) have been described, particularly in exon 7 and 8 [90,91].

*HNF4A* heterozygous mutations usually cause haploinsufficiency that impairs insulin secretion and causes HNF4A-MODY (MODY1) [102,103,104] with the same mechanisms described above for *HNF1A* mutations [133]. Of note, HNF4A regulates the expression of *HNF1A* [134] and is therefore expected that, with the exception of normoglycemic glycosuria [135], the HNF4A-MODY phenotype is similar to that of HNF1A-MODY, with glucose intolerance becoming evident during adolescence or early adulthood and deteriorating with age [105]. Chronic diabetes complications are frequent and their development is related to the degree of metabolic control [107,136,137]. In addition to their effects on β-cell function, HNF4A deficiencies affect liver function, triglyceride serum levels (50% reduction) and apolipoprotein biosynthesis (25% reduction of apolipoproteins AII and CIII and Lp(a) lipoprotein serum concentrations). Reduced HDL and increased LDL cholesterol levels have been also described [138,139] in patients with HNF4A-MODY. 

#### HNF4A Actionability

As described for carriers of *HNF1A* mutations, patients with HNF4A-MODY are also sensitive to low-dose sulfonylureas. In fact, HNF4A-MODY patients can initially be treated with diet while sulfonylureas are recommended if glycemic control deteriorates [107]. Especially in young adults, this treatment results in better glycemic control than that achieved with insulin [102]. If hypoglycemia is not a problem, this treatment can be maintained for decades [116,140]. A short-acting agent such as a meglitinide can be considered in children [118,119,120]. 

An alternative treatment for HNF4A-MODY patients are GLP-1 agonists that should be taken into consideration in case of either non-optimal glycemic control or recurrent hypoglycemia due to sulfonylureas [122,126].

Given the very high risk of massive macrosomia (birth weight > 5000 g) and related clinical outcome of babies delivered by women with *HNF4A*-MODY, tight maternal glycemic control is instrumental [69,131,141]. 

Of note, 15% of newborns with *HNF4A* mutations have diazoxide-responsive neonatal HH which remits during infancy [131,141,142].

### 5.3. HNF1B 

*HNF1B* encodes the hepatocyte nuclear factor 1β (HNF1B), a transcription factor with 80% homology with HNF1A, which binds DNA either as a homodimer or a heterodimer with HNF1A. Heterozygous mutations (e.g., missense mutations, complete gene deletions, little fragment deletions or insertions), half of which are de novo [143], show autosomal dominant inheritance and cause a clinical spectrum with renal cysts and hyperglycemia, (HNF1B-MODY, MODY5). HNF1B is a rare cause of MODY, accounting for <2% of cases [144]. In HNF1B-MODY hyperglycemia is usually diagnosed in the young adulthood (mean age of 26 with a range of 10–61) [145]. The range of glucose homeostasis goes from normoglycemia to insulin-treated diabetes with ketoacidosis [146]. Only few cases of neonatal diabetes have been described in carriers of *HNF1B* mutations [147,148].

The combination of diabetes with congenital anomalies in the kidney (cysts) and urinary tract is the most consistent clinical presentation in individuals with *HNF1B* mutations [149,150,151,152,153]. Alterations in pancreas, brain, parathyroid gland, and female genital tract (e.g., rudimental uterus, vaginal aplasia, bicornuate uterus and double vagina) are frequently described and are compatible with the involvement of HNF1B in their development [146,154,155,156,157,158].

Liver impairment can also manifest as neonatal jaundice, lack of intrahepatic bile ducts, rise of alanine aminotransferase and γ-glutamyl transferase serum levels [159]. Hypercholesterolemia and hyperuricemia have been also described [160,161]. 

#### HNF1B Actionability

*HNF1B* mutation carriers have insulin deficiency due to pancreatic hypoplasia [154]. Therefore, not unexpectedly, these patients require early insulin therapy [162,163] and do not/or rarely respond adequately to sulfonylureas [164]. 

Moreover, patients with *HNF1B* mutation have reduced exocrine pancreatic function that should, therefore, be monitored by measuring fecal elastase levels [165]. Uric acid levels should also be monitored in order to prevent gout. Systematic screening for all potential morphological anomalies, in particular for renal cysts and pancreatic and genital (especially in females) abnormalities [88], should be made. 

During pregnancy, women with HNF1B-MODY typically require insulin for glycemic control. Generally, because of maternal hyperglycemia, birth weight of their babies is increased [166]. Conversely, when the fetus has a *HNF1B* mutation (e.g., inherited from the father) birth weight can be diminished reflecting reduced insulin secretion in the uterus [166].

## 6. PPARG 

The Peroxisome proliferator-activated receptor γ (*PPARG*) gene encodes a ligand-activated transcription factor (PPARγ) belonging to the family of nuclear PPARs. PPARγ is predominantly expressed in white and brown adipose tissue [167,168,169] and exists as two isoforms, PPARγ1 and PPARγ2, with the latter containing an additional 30 amino acids at its N-terminus [169,170]. PPARγ is a key regulator of adipocyte differentiation as well as a potent modulator of whole-body energy balance, lipid biosynthesis, and insulin sensitivity [171,172]. Several SNPs and/or rare variants and mutations have been associated with a spectrum of metabolic diseases including obesity, syndromic form of monogenic diabetes and type 2 diabetes [173,174,175,176]. 

Heterozygous LOF mutations of *PPARG* are associated with a familial partial lipodystrophy type 3 (FPLD3) considered as a monogenic model of the common “metabolic syndrome” [177,178]. FPLD3 is a dominantly inherited syndrome, characterized by specific loss of subcutaneous fat from the limbs and gluteal region. It is also associated with early diabetes onset, hypertension, severe insulin resistance, extreme dyslipidemia, and hepatic steatosis [177,179]. The severity of lipodystrophy is related to the deleterious effect of each specific mutation on PPARγ function [180].

To date approximately 40 FPLD3 LOF *PPARG* mutations have been reported, most of which involving the ligand-binding domain or the DNA-binding domain [173,180]. Mechanisms of negative dominance and haploinsufficiency have both been suggested to explain the pathogenicity of *PPARG* mutations [181]. Notably, approximately 0.2% of the general population carries a rare missense variant in *PPARG* gene, 20% of which are functionally relevant and are associated with metabolic diseases, not necessarily leading to overt FPLD3 [182]. These evidences support the hypothesis that additional mechanisms such as gene-gene and gene-environment interactions might contribute to the variable phenotype [173].

### PPARG Actionability

In 1995, thiazolidinediones (TZDs) have been identified as a class of potent activators of PPARγ, able to promote adipogenesis and improve insulin sensitivity [183,184,185,186]. Due to their ability to preserve pancreatic β-cell function and reduce insulin resistance, TZDs have become an established medication for type 2 diabetes [187]. Unfortunately, data on the use of TZDs in individuals with monogenic syndromic form of diabetes caused by LOF mutations in *PPARG* are still inconclusive. This is partly due to the rarity of this form, but also to the heterogeneous pharmacological response, probably secondary to the specific molecular defect [188,189,190]. At this regard, there is some evidence suggesting that knowing the specific molecular defect in a given patient may be helpful in choosing the most appropriate TZD [188,191]. For example, patients carrying the R308P mutation in PPARγ show reduced binding affinity to rosiglitazone, but not to pioglitazone [192].

Overall, when TZD is able to bind a mutated PPARγ some metabolic improvement including reductions of glycated hemoglobin, triglycerides, free fatty acids and increased body fat has been described [189,190,192,193]. 

Therefore, with due care and waiting for further and larger studies possibly testing also new TZD-derived compounds, *PPARG* can be envisioned as a likely “actionable gene” for patients with FPLD3 [194].

## 7. GATA4 and GATA6 

GATA family includes a group of six transcription factors (GATA1–6), all of which contain two tandem zinc-finger domains that bind a consensus site (A/T) GATA (A/G) of DNA target region and play a crucial role in the development and differentiation of all eukaryotic organisms [195].

LOF in *GATA4* and *GATA6* are associated with pancreatic agenesis/hypoplasia and diabetes, along with congenital heart abnormalities and several cancers [196].

### 7.1. GATA4

*GATA4* heterozygous LOF mutations and complete deletion of the gene (deletion from 1 to 17 Mb that includes 8 additional genes), have been associated with congenital heart malformations [197,198,199], and more rarely with pancreatic agenesis and neonatal diabetes [200,201]. 

### 7.2. GATA6

*GATA6* heterozygous mutations (e.g., missense, non-sense, frameshift mutations and small deletions mainly located in, or near to, tandem zinc-finger domains or in the splicing site), most of which occur de novo, can cause a wide spectrum of diabetes manifestations, ranging from pancreatic agenesis and neonatal diabetes to adult-onset diabetes. Usually, age at diabetes diagnosis ranges from 12–46 years [202]. In addition, exocrine pancreatic insufficiency and, more frequently, congenital heart malformations, together with congenital biliary tract anomalies, gut developmental disorders, neurocognitive abnormalities and additional endocrine abnormalities have been described [203]. Sometimes, the same mutation has been associated with alternative phenotypes showing a dissimilar penetrance in different patients [204]. No data in pregnant women are available, while low birth weight has been reported for babies carrying *GATA4* or *GATA6* mutations [201,202].

### 7.3. GATA4 and GATA6 Actionability

For these two genes, actionability consists in the early identification of the possible severe abnormalities mutations carriers can show. In fact, in *GATA4/6* mutations carriers, particular in newborns, the evaluation and monitoring over time of congenital heart malformations and heart failure is mandatory. As a consequence of pancreatic agenesis/hypoplasia, these patients not only require insulin as the treatment of choice for hyperglycemia but may also need pancreatic enzyme supplementation. Since the same *GATA4/6* mutations can generate variable phenotypes in different subjects, a case-by-case evaluation is necessary. 

## 8. Conclusions

There is no doubt that a correct, precise and timely diagnosis is essential for the rapid management of most diseases and for the prevention of related complications. This general assumption also often applies when dealing with genetic diseases [205], thus stressing that timely recognition of patients who deserve genetic testing is extremely important. 

Hopefully this article makes it clear that some forms of monogenic diabetes are optimal examples of precision medicine and of translating research findings into clinical practice. Rare forms of monogenic diabetes due to mutations of “actionable genes” have, in fact, paved the way for new therapeutic strategies and specific management of disease outcomes. Clinical diabetologists should, therefore, carefully consider who of their patients deserves timely genetic testing for monogenic diabetes, possibly using NGS approaches. Of note, the advent of NGS not only has allowed a prompt diagnosis but also has increased the chance of discovering new “diabetes genes” [206,207], a prerequisite to understand in depth glucose homeostasis and to possibly invent new drugs. It is conceivable that some of the genes we will discover in the future will be “actionable”, thus further increasing the number of patients who can be specifically managed according to their specific genetic defects.

## Figures and Tables

**Table 1:** Summary of actionable genes with related diabetes phenotype and actionability.

Gene	Mutation	Phenotype	Disease Mechanism	Additional Complication	Birth Weight	Actionability
*ABCC8/* *KCNJ11*	Heterozygous and Homozygous GOF	Neonatal diabetes MODY Adult-onset diabetes	K_ATP_ channel permanently open, K^+^ efflux/membrane hyperpolarization/defective insulin secretion	Neurodevelopment dysfunction	Normal, as long as maternal hyperglycemia is properly treated Low, when only the fetus is mutated	High dose of sulphonylureas (also in pregnancy, as long as the fetus is mutated; otherwise, insulin should be given)
*GCK*	Heterozygous LOF Homozygous/Compound heterozygous LOF	GCK-MODY (moderate fasting hyperglycemia from birth, low risk of chronic complication) Neonatal diabetes	Increased glucose sensor threshold (glucose stimulated insulin secretion begins at higher glucose level)	None	Normal, as long as maternal hyperglycemia is properly treated Low, when only the fetus is mutated	No treatment needed (except during pregnancy when insulin is the treatment of choice)
*HNF1A*	Heterozygous and Homozygous LOF	HNF1A-MODY (fasting glycemia increase with age, normoglycemic glycosuria, liver adenomatosis)	Reduced HNF1A expression, reduced β-cell mass, blunted glycolysis and ATP production and eventually defective insulin secretion	Retinopathy, nephropathy and neuropathy are common. Ketoacidosis can develop	Normal, as long as maternal hyperglycemia is properly treated	Low dose of sulphonylureas also in pregnancy for the first two trimesters (when both the mother and the fetus are mutated)
*HNF4A*	Heterozygous LOF	HNF4-MODY (fasting glycemia increase with age, liver dysfunction)	Reduced HNF1A expression, reduced β-cell mass, blunted glycolysis and ATP production and eventually defective insulin secretion	Reduced triglycerides and lipoprotein serum concentration	Normal, as long as maternal hyperglycemia is properly treated	
*HNF1B*	Heterozygous LOF	HNF1B-MODY (high fasting glycemia, ketoacidosis)	Reduced HNF1B expression, pancreatic hypoplasia, blunted glycolysis and ATP production and eventually defective insulin secretion	Kidney cysts and urinary tract abnormalities, atrophic pancreas, genital abnormalities, hyperuricemia, gout	Normal, as long as maternal hyperglycemia is properly treated Low, when only the fetus is mutated	Systemic screening for renal cysts, exocrine pancreatic function and genital abnormalities (especially in females)
*PPARG*	Heterozygous LOF	Severe insulin resistance	Defective adipocyte differentiation due to PPARG haploinsufficiency or dominant negative LOF mutation	Familial partial lipodystrophy type 3 (early-onset diabetes, hypertension, severe insulin resistance and dyslipidemia, hepatic steatosis)	No clear data are available	Thiazolidinediones
*GATA4*	Heterozygous LOF or complete gene deletion	Neonatal diabetes	Dysfunctional transcriptional activity, and altered embryonic organ development	Congenital heart malformation, pancreatic agenesis or hypoplasia	Low	Evaluation and follow up of congenital heart malformation and pancreatic agenesis/hypoplasia
*GATA6*	Heterozygous LOF	Neonatal diabetes Adult-onset diabetes	Dysfunctional transcriptional activity, and altered embryonic organ development	Congenital biliary tract anomalies, gut developmental disorders, neurocognitive abnormalities, additional endocrine abnormalities	Low	

GOF: gain of function. LOF: loss of function.

## Data Availability

Not applicable.
